# Morphometric MRI as a diagnostic biomarker of frontotemporal dementia: A systematic review to determine clinical applicability

**DOI:** 10.1016/j.nicl.2018.08.028

**Published:** 2018-08-31

**Authors:** Jillian McCarthy, D. Louis Collins, Simon Ducharme

**Affiliations:** McConnell Brain Imaging Centre, Montreal Neurological Institute, McGill University, Montreal, Canada

**Keywords:** Frontotemporal dementia, Classification, MRI, Morphometric analysis, Machine learning, Diagnostic biomarker, FTD, frontotemporal dementia, bvFTD, behavioral variant frontotemporal dementia, AD, Alzheimer's disease, C, Controls, PPA, primary progressive aphasia, nfvPPA, nonfluent variant PPA, svPPA, semantic variant PPA, lvPPA, logopenic variant PPA, DLB, dementia with Lewy Bodies, VaD, vascular dementia, SVM, support vector machines, CV, cross-validation, LOOCV, leave-one-out cross-validation, VBM, voxel-based morphometry, DWI, diffusion weighted imaging, DTI, diffusion tensor imaging, GM, gray matter, WM, white matter, ROI, region of interest, Acc, accuracy, SS, sensitivity, SP, specificity, AUC, Area under a receiver operator characteristic curve, DBM, deformation-based morphometry, TBM, tensor-based morphometry, PCA, principle component analysis, NDH, Network Degeneration Hypothesis, WMH, white matter hyperintensities, L, left, R, right, B, bilateral, RD, radial diffusivity, FA, fractional anisotropy, MD, mean diffusivity, AD, axial diffusivity, LD, longitudinal diffusivity, TD, trace diffusivity, MTL, medial temporal lobe, DLPFC, dorsolateral prefrontal cortex, VMPFC, ventromedial prefrontal cortex, CT, cortical thickness, Differential-STAND, Differential Diagnosis Based on Structural Abnormality due to Neurodegeneration (Vemuri et al., 2011), LoCo, Loss in Connectivity (the percent of WM tracts out of the total connecting to a GM region in a normal control that pass through voxels identified in a WM “injury” map (Kuceyeski et al., 2012)

## Abstract

Frontotemporal dementia (FTD) is difficult to diagnose, due to its heterogeneous nature and overlap in symptoms with primary psychiatric disorders. Brain MRI for atrophy is a key biomarker but lacks sensitivity in the early stage. Morphometric MRI-based measures and machine learning techniques are a promising tool to improve diagnostic accuracy. Our aim was to review the current state of the literature using morphometric MRI to classify FTD and assess its applicability for clinical practice. A search was completed using Pubmed and PsychInfo of studies which conducted a classification of subjects with FTD from non-FTD (controls or another disorder) using morphometric MRI metrics on an individual level, using single or combined approaches. 28 relevant articles were included and systematically reviewed following PRISMA guidelines. The studies were categorized based on the type of FTD subjects included and the group(s) against which they were classified. Studies varied considerably in subject selection, MRI methodology, and classification approach, and results are highly heterogeneous. Overall many studies indicate good diagnostic accuracy, with higher performance when differentiating FTD from controls (highest result was accuracy of 100%) than other dementias (highest result was AUC of 0.874). Very few machine learning algorithms have been tested in prospective replication. In conclusion, morphometric MRI with machine learning shows potential as an early diagnostic biomarker of FTD, however studies which use rigorous methodology and validate findings in an independent real-life cohort are necessary before this method can be recommended for use clinically.

## 1 Introduction

Frontotemporal dementia (FTD^1^) is one of the most common forms of early onset dementia, occurring with similar frequency to Alzheimer's Disease (AD) in people under the age of 65 (Onyike and Diehl-Schmid, 2013). This heterogeneous disorder most often presents with combinations of personality changes such as apathy, loss of empathy, and disinhibition (behavioral variant – bvFTD) (Rascovsky et al., 2011) or language deficits (primary progressive aphasia – PPA). PPA is further divided into three variants - semantic (svPPA), nonfluent (nfvPPA) and logopenic (lvPPA) (Gorno-Tempini et al., 2011). The pathology underlying frontotemporal lobar degeneration is equally heterogeneous and involves abnormal accumulation of proteins including microtubule-associated protein tau, transactive response DNA-binding protein with molecular weight 43 kDa (TDP-43), and fused in sarcoma (FUS) protein, while the lvPPA clinical syndrome is most commonly associated with AD pathology (Rademakers et al., 2012).

The diagnosis of FTD currently poses a significant challenge for clinicians as the presenting symptoms overlap considerably with other diseases including primary psychiatric disorders and other dementias (Ducharme et al., 2015). This is especially true of bvFTD. Evidence suggests as many as 50% of people with bvFTD are initially diagnosed with a psychiatric disorder (Woolley et al., 2011). As well, significant memory impairment can exist in bvFTD, comparable to that seen in AD (Bertoux et al., 2014; Mansoor et al., 2015).

The most common imaging method currently used in clinical practice is structural MRI, which is insufficiently sensitive for early stage diagnosis of FTD given that atrophy can be very subtle at the disease onset. Indeed, in a mixed neuropsychiatric population that is representative of clinical practice, a standard MRI with visual review had insufficient sensitivity (70%) to identify cases with bvFTD, while the usual alternative of [18F] FDG-PET had poor specificity (68%) (Vijverberg et al., 2016). This can lead to erroneous or significantly delayed diagnosis, causing prolonged periods of uncertainty for patients and their families. The development of improved diagnostic biomarkers for the early detection of FTD is critical to ensure patients are getting the appropriate care as well as for the accurate identification of patients for clinical trials. Improving MRI methods is ideal given that MRI is already part of standard practice and there are currently no validated molecular biomarkers for FTD diagnosis. AD cerebral spinal fluid (CSF) and PET amyloid tracers can be used in the differential diagnosis of FTD from AD, as FTD will likely be negative for these (Meeter et al., 2017), however FTD-specific CSF biomarkers or tau tracers are not available.

There has been considerable interest in automated morphometric analysis of MRI, most commonly assessing gray matter (GM) atrophy and, in recent years, white matter (WM) integrity using diffusion tensor imaging (DTI). Techniques such as voxel-based morphometry (VBM) and cortical thickness have demonstrated specific patterns of frontal and temporal GM atrophy on a group level (Meeter et al., 2017). These patterns differ from those seen in other dementias (such as hippocampal atrophy found in AD). BvFTD is associated with atrophy primarily in the frontal lobe, insula, anterior cinguate cortex and basal ganglia (Meeter et al., 2017; Pan et al., 2012; Schroeter et al., 2014). PPA is primarily associated with left-sided atrophy (language dominant hemisphere) in the initial disease stages; nfvPPA with inferior frontal and insular atrophy, svPPA with anterior temporal atrophy, and lvPPA with posterior temporal and parietal atrophy (Bisenius et al., 2016; Meeter et al., 2017; Mesulam et al., 2009; Rogalski et al., 2014). WM changes have a more widespread distribution and likely precede GM atrophy (Lam et al., 2014; Mahoney et al., 2014; Meeter et al., 2017).

A high discriminative power is needed to differentiate between diseases on an individual level, in order to be useful in clinical practice. However, with improving methods of morphometric analysis and the use of multivariate statistics and machine learning methods, it is becoming increasingly feasible to improve diagnosis at the individual level. An extensive body of literature exists classifying AD in this way. These studies have found overall high accuracy levels when comparing AD to controls (often >90% accuracy) (Falahati et al., 2014; Rathore et al., 2017). In recent years several studies have attempted this type of classification for the diagnosis of FTD using a variety of MRI measures and machine learning algorithms.

The aim of this systematic review is to summarize the current literature studying the diagnostic classification of FTD utilizing morphometric MRI data on an individual level, with the aim of evaluating its potential usefulness and readiness for clinical practice.

## 2 Method

This systematic review follows the recommendations of PRISMA (McInnes et al., 2018; Moher et al., 2009) as applicable. An initial search was conducted up to March 12, 2018 using PubMed and PsychINFO with the following search terms: (frontotemporal dementia OR frontotemporal lobar degeneration) AND MRI AND ((diagnostic OR diagnosis) AND (accuracy OR classification OR prediction)). The search was limited to peer-reviewed, full text articles, published in English within the last 10 years (2007 or later) to focus on the most advanced image processing methods. All resulting papers were screened by title and abstract to exclude irrelevant studies, and full texts of selected articles were reviewed. Studies were included if they meet the following criteria: (1) conducted a diagnostic classification of FTD (behavioral or language variant, or both variants combined) versus controls or versus other disorders on an individual subject level and (2) used classification features derived from structural MRI, either alone or in combination. In the case of studies which conducted classifications based on MRI morphometry alone and in combination with other methods, only those results pertaining to MRI morphometry were included in this review. Reference lists of included articles were also manually searched to identify other relevant articles. The risk of bias and applicability of each included study was assessed with the QUADAS-2 tool (Whiting et al., 2011).

## 3 Results

**Fig. 1:**
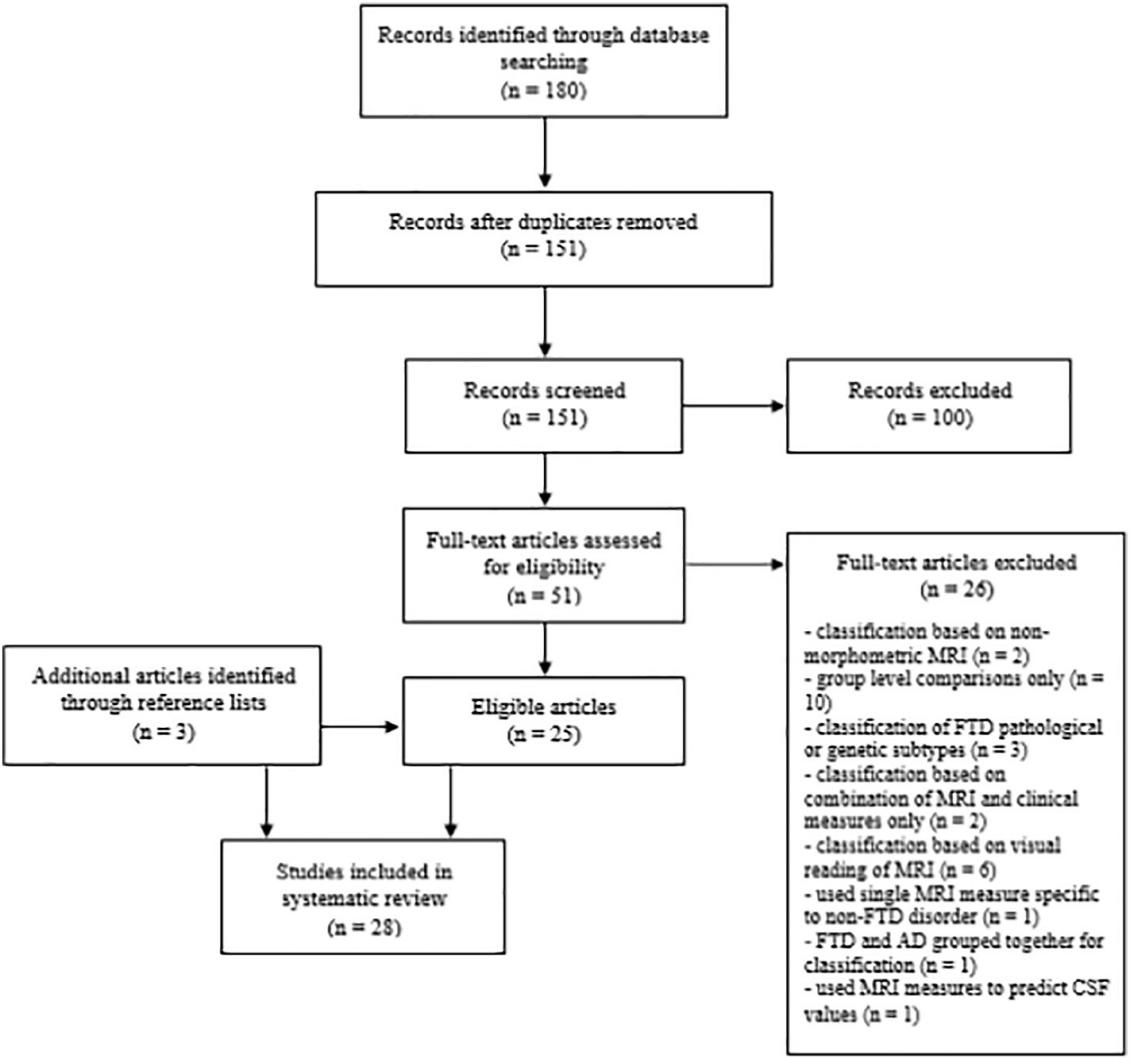
PRISMA flow chart of study selections.

The search produced 151 articles. Of these, 25 relevant articles were identified. Cross-reference list searches of each relevant article yielded three additional papers, resulting in a total of 28 papers for inclusion in this review (Fig. 1).

### 3.1 Study characteristics

Eleven studies conducted a binary classification of FTD or specifically bvFTD from a control group. Seventeen studies conducted a binary classification of FTD or specifically bvFTD from AD. Six studies conducted a multi-class classification to differentiate FTD, AD and controls, while four studies conducted a multi-class classification between various dementia types and controls. Four studies conducted classifications of PPA; two studies differentiated PPA subtypes from each other and controls. One study classified PPA from controls. One study differentiated FTD subtypes (bvFTD and PPAs) from a combined group of all other subtypes and AD. Results are summarized in Table 1, Table 2, Table 3, Table 4, Table 5. Accuracy, sensitivity, specificity, and/or area under the receiver operating characteristic curve (AUC) are reported, if provided. In cases where raw numbers were reported, applicable performance measures were calculated from these numbers. In this paper we consider performance of 90% or greater as high, 70–90% as moderate, and <70% as low.

**Table 1:** Classifications of bvFTD versus Controls or AD.

bvFTD						vs Controls				vs AD			
Name	Sample	Classification		Measure	ROIs	Acc	SS	SP	AUC	Acc	SS	SP	AUC
Canu et al., 2017	27 bvFTD	Random forest		Cortical thickness	L inferior parietal Best 5 (L inferior parietal, R temporal pole, L isthmus cingulate, R inferior parietal, R precuneus)					78	76	83	
	62 AD				Best 5 (L inferior parietal, R temporal pole, L isthmus cingulate, R inferior parietal, R precuneus)					82	80	87	
				DWI	R uncinate; AD					81	96	43	
					Best 5 (R uncinate; AD, RD, MD, FA, Genu of CC; FA)								
										81	89	61	
				Combination	5 CT + 5 WM tract					82	76	96	
					Best 5 (L inferior parietal, R temporal pole, R precuneus, L isthmus cingulate, L superior parietal)					84	79	81	
Chow et al., 2008	16 bvFTD	Logistic regression		Volumes	L medial middle frontal parenchymal	87	68.8	96.6					
	30 C												
Frings et al., 2014	15 bvFTD	Logistic regression		Volume	caudate					79			
					caudate + gyrus rectus GM					83			
	14 AD												
Mahoney et al., 2014	27 bvFTD			DTI-RD	Whole-brain		82	80	0.82				0.67
	25 AD				Corpus callosum		93	75	0.85				
					L uncinate fasciculus		82	75	0.82				
					L cingulum bundle		74	70	0.83				
	20 C												
				DTI-FA	Whole-brain				0.73		78	68	0.74
					L uncinate fasciculus						77	68	0.76
					L cingulum bundle						63	80	0.67
					Corpus callosum						56	80	0.73
				DTI-TD	Whole-brain				0.80				0.66
				DTI-AD	Whole-brain				0.74				0.59
Meyer et al., 2017	52 bvFTD	SVM		VBM-GM density	Whole-brain	81.7	78.9	84.6					
		LOOCV			Frontal lobe	80.7	76.9	84.6					
					Frontal + Basal ganglia & insula	82.7	80.7	84.6					
	52 C												
					Temporal lobe	78.8	76.9	80.8					
					Frontal & temporal lobe	84.6	80.7	88.5					
					Frontal + Temporal + Basal Ganglia & insula	84.6	80.7	88.5					
Möller et al., 2016	26 bvFTD	SVM	Training Set LOOCV	VBM-GM density	Whole-brain	75	62	83		81	69	88	
	42 AD												
	47 C												
	25 bvFTD		Test Set			85	60	98	0.87	82	64	93	0.81
	42 AD												
	47 C												
Raamana et al., 2014	30 bvFTD	SVM	LOOCV	Surface displacements	L Hippocampus		14	83	0.488		37	62	0.492
					R Hippocampus		43	83	0.631		50	41	0.456
					L lateral ventricle		79	87	0.826		60	82	0.712
	34 AD												
					R lateral ventricle		64	87	0.755		63	79	0.714
	14 C												
			Train/Test		L Hippocampus		50	62	0.562		50	56	0.528
					R Hippocampus		25	75	0.5		0	1	0.5
					L lateral ventricle		100	88	0.938		75	56	0.653
					R lateral ventricle		75	100	0.875		62	67	0.646
Wang et al., 2016	55 bvFTD	Naïve Bayes		VBM-GM volume	Amygdale, hippocampus, MTL, temporal pole, DLPFC, VMPFC, striatum and insula					51.4	36.4	66.7	
	54 AD	10-fold CV

**Table 2:** Classifications of FTD vs Controls or AD.

FTD					vs Controls				vs AD			
Name	Sample	Classification	Measure	ROIs	Acc	SS	SP	AUC	Acc	SS	SP	AUC
Bron et al., 2017	33 FTD	SVM	VBM-GM volume	Whole-brain				0.95				0.78
	24 AD	4-fold CV	VBM-WM volume					0.96				0.76
	34C		VBM-Supratentorial brain volume					0.95				0.72
			DTI-FA									
			VBM-WM					0.91				0.80
			volume + DTI-FA					0.95				0.81
Davatzikos et al., 2008	12 FTD	SVM	RAVENS-GM and WM volume	PCA	100				84.3			
	37 AD	LOOCV										
	12 C	Fisher's discriminant Analysis	Volume	Hippocampal, ventricular, total brain	75				70.9			
Du et al., 2007	19 FTD	Logistic regression	Volume	Frontal	89							
	22 AD	LOOCV		Parietal	81				79			
	23 C			Temporal	85							
			Cortical thickness	Frontal	88							
				Parietal	82				82			
				Temporal	85							
Dukart et al., 2011	14 FTD	SVM	GM	Whole-brain	77.8				80			
	21 AD	LOOCV	WM	Whole-brain	77.8				74.3			
	13C											
			GM	ROI (a priori)	85.2				60			
Klöppel et al., 2008b	19 FTD	SVM	GM volume	Whole brain					89.2	94.7	83.3	
	18 AD	LOOCV										
Klöppel et al., 2015	12 FTD	SVM	VBM-GM volume	Whole-brain								0.78
	122 AD	Separate test set										
Lehmann et al., 2010	23 FTD	SVM	Cortical Thickness	Whole-brain					79.4	91.3	54.5	0.87
	17 AD	2-level CV										
McMillan et al., 2012	38 FTD	Logistic regression	GM density	Precuneus						82	79	0.883
				Posterior cingulated						87	66	0.890
				Anterior temporal						79	69	0.792
	29 AD											
			DTI-FA	Corpus callosum						79	59	0.795
			Combination	Corpus callosum, precuneus, posterior cingulated						87	83	0.938
McMillan et al., 2014	72 FTD	Linear regression	Cortical thickness	Data-driven						89	81	0.778
				Anatomical						100	54	0.802
	21 AD	Train/test										
			Volume	Global GM						65	100	0.820
				Global ventricles						100	65	0.826
			DTI-FA	Data-driven						100	46	0.808
				Anatomical						56	78	0.649
			Combination	Data-driven						89	89	0.874
				Anatomical						78	70	0.742
Muñoz-Ruiz et al., 2012	37 FTD	Regression	Volume	Hippocampus	83	80	84		55	55	55	
	46 AD	Train/Test										
	26 C											
			TBM	Hippocampus, amygdala, posterior temporal lobe, lateral ventricle in frontal horn, central part and occipital horn, lateral ventricle in temporal horn, gyri hippocampalis et ambiens, anterior cingulate gyrus and superior frontal gyrus.	82	90	77		62	67	56	
			VBM-GM concentration		83	91	77		72	76	67	
			VBM-GM volume		85	89	82		69	71	66	
Whitwell et al., 2011	14 FTD	Logistic regression	GM volume	Temporoparietal cortex								0.81
				Hippocampus								0.74
				Temporoparietal cortex + hippocampus								0.93
	14 AD											
Zhang et al., 2013	25 FTD	Logistic regression	VBM-GM volume	ROI1 (B frontotemporal, anterior callosal)	65.7	80.1	48.7	0.665				
			
	19 C	4-fold CV		ROI2 (L temporal)	63.9	77.0	46.6	0.722				
				ROI3 (L dorsal frontal)	45.7	74.2	5.4	0.566				
			VBM-WM volume	ROI1	59.2	77.2	34.6	0.627				
				ROI2	58.1	71.5	36.4	0.657				
				ROI3	47.4	79.8	5.3	0.606				
			DTI-RD	ROI1	76.0	79.9	72.3	0.853				
				ROI2	81.4	80.7	80.5	0.877				
				ROI3	67.6	73.3	58.6	0.722

**Table 3:** Multi-class Classifications of FTD, AD, and Controls.

FTD, AD and controls								
Name	Sample	Classification	Measure	ROIs	Acc	SS (FTD)	SP (FTD)	AUC
Bron et al., 2017	33 FTD	SVM	VBM-GM volume	Whole-brain				0.85
	24 AD	4-fold CV	VBM-WM volume					0.83
	34 C		VBM-Supratentorial brain volume					0.84
			DTI-FA					0.83
			VBM-WM volume + DTI-FA					0.87
Dukart et al., 2011	14 FTD	SVM	GM	Whole-brain	72.9			
	21 AD	LOOCV	WM		66.7			
	13 C							
			GM	a priori ROIs	56.3			
Kuceyeski et al., 2012	18 FTD	Linear discriminant analysis	GM volume	Whole-brain parcellation	76.36	81.08	66.67	
			DWI-FA		76.36	72.97	83.33	
			DWI-RD		89.09	97.30	72.22	
			DWI-LD		85.45	89.19	77.78	
			Combination GM + DWI		83.64	91.89	66.67	
			LoCo		87.27	91.89	77.78	
	18 AD							
		LOOCV						
	19 C							
Möller et al., 2015	30 bvFTD	Discriminant function analyses LOOCV	1st analysis: VBM-GM volume, Subcortical volumes, DWI-FA	Significant voxels/regions from paired group comparisons	91.4	66.7		
	39 AD							
	41 C							
			2nd analysis: VBM-GM volume, subcortical volumes, DWI- AD, DWI-RD		86	75		
Raamana et al., 2014	30 bvFTD	SVM	Volumes	L Hippocampus				0.5
	34 AD	Train/Test		R Hippocampus				0.54
				L lateral ventricle				0.5
	14 C			R lateral ventricle				0.5
			Laplacian invariants	L Hippocampus				0.5
				R Hippocampus				0.49
				L lateral ventricle				0.5
				R lateral ventricle				0.59
			Surface displacements	L Hippocampus				0.66
				R Hippocampus				0.56
				L lateral ventricle				0.76
				R lateral ventricle				0.77
Wang et al., 2016	55 bvFTD	Naïve Bayes	VBM-GM volume	Amygdale, hippocampus, MTL, temporal pole, DLPFC, VMPFC, striatum and insula	54.2			
	54 AD	10-fold CV						
	57 C

**Table 4:** Multi-class Classifications of Dementia.

Multi dementia types								
Name	Sample	Classification	Measures	ROIs	Acc	SS (FTD)	SP (FTD)	AUC (FTD)
Klöppel et al., 2015	12 FTD	SVM	VBM-GM volume	Whole-brain				0.78
	122 AD	Separate test cohort						
	4 DLB							
	18C							
Koikkalainen et al., 2016	92 FTD	Disease State Index (DSI)	Volumes	Whole-brain parcellation	50.4			
			VBM-GM concentration		65.1			
		10-fold CV	TBM		64.3			
			Manifold learning	Hippocampus and frontotemporal lobe	50.4			
			ROI-based grading		58.3			
			Vascular burden- WMH, cortical and lacunar infarcts volumes		32.7			
	223 AD							
			All features		70.6	62	95	
	47 DLB							
	24 VaD							
	118 C							
Tong et al., 2017	92 FTD	RUSBoost	Volumes	Whole-brain parcellation	58.6			
					66.6			
					70			
		10-fold CV	Grading					
			Combination					
	219 AD							
	47 DLB							
	24 VaD							
	118 C							
Vemuri et al., 2011	47 FTD	Differential-STAND	GM density	Whole brain		84.4	93.8	
	48 AD	LOOCV						
	20 DLB							
	21 C

**Table 5:** PPA classifications.

Name	Sample	Classification	Measures	ROIs	Acc	SS	SP	AUC	Acc	SS	SP	AUC	Acc	SS	SP	AUC
					nfvPPA vs Controls				lvPPA vs Controls				svPPA vs Controls			
Bisenius et al., 2017	16 nfvPPA	SVM	VBM-GM density	Whole-brain ROI (a priori from meta-analyses)	91	88	94	0.94	95	91	100	0.95	97	94	100	0.97
					84	81	88	0.90	82	82	82	0.91	100	100	100	1
	17 svPPA	LOOCV														
	11 lvPPA															
	20 C															
Wilson et al., 2009	32 nfvPPA	SVM	GM volume	PCA	89.1	87.5	90.6	0.941	100	100	100	1	100	100	100	1
	38 svPPA	2-level CV														
	16 lvPPA															
	115 C															

					svPPA vs nfvPPA				lvPPA vs svPPA				lvPPA vs nfvPPA			
Bisenius et al., 2017	16 nfvPPA	SVM	VBM-GM density	Whole-brain ROI (a priori from meta-analyses)	78	81	75	0.88	95	100	91	0.93	55	64	45	0.59
	17 svPPA	LOOCV														
					78	81	75	0.87	95	100	91	0.91	64	73	55	0.64
	11 lvPPA															
	20 C															
Wilson et al., 2009	32 nfvPP	SVM	GM volume	PCA	89.1	84.4	93.8	0.964	93.8	93.8	93.8	0.984	81.3	81.3	81.3	0.879
	38 svPP	2-level CV														
	16 lvPPA															
	115 C															

					PPA (svPPA and nfvPPA) vs Controls											
					*Acc*				*SS*				*SP*			
Chow et al., 2008	14 PPA	Logistic regression	Volumes	L anterior temporal	90.9				78.6				96.7			
	30 C															

					bvFTD vs others				svPPA vs. others				nfvPPA vs others			
					*Acc*	*SS*	*SP*		*Acc*	*SS*		*SP*	*Acc*	*SS*		*SP*
Tahmasian et al., 2016	11 bvFTD	SVM	VBM-GM volume	A priori based on the NDH	72.5	45.4	82.7		92.5	50		97.5	82.5	0		94.2
	4 svPPA	LOOCV														
	5 nfvPPA															
	20 AD															

Studies varied considerably in methodology. The majority of studies looked at changes in GM structure, most commonly using VBM to assess either GM concentration or volume. WM integrity was commonly assessed using DTI measures. Studies used a variety of whole-brain and region of interest (ROI) based approaches, including a priori selection of ROIs and the use of ROIs that showed significant differences in group-level comparison. Studies also varied widely in classification methods. Machine learning classification techniques were utilized by most studies, the most common being support vector machines (SVM). Most studies used a k-fold cross validation (CV) approach, most commonly with a leave-one-out CV strategy. Only one study used independent subject data (from a different cohort) in a separate testing set (Klöppel et al., 2015).

Almost all studies used a clinically defined diagnosis as the reference standard. Six studies (Chow et al., 2008; Frings et al., 2014; Mahoney et al., 2014; Meyer et al., 2017; Muñoz-Ruiz et al., 2012; Wang et al., 2016) included a subset of patients with pathologically confirmed diagnosis or those with a known genetic mutation consistent with FTD. Three studies (Klöppel et al., 2008b; Lehmann et al., 2010; Vemuri et al., 2011) used pathologically defined dementia diagnosis as the gold standard. Two studies (McMillan et al., 2014; McMillan et al., 2012) grouped subjects as AD or FTD based on the presence or absence of CSF biomarkers consistent with AD. Studies also varied considerably in disease severity. Studies report a variety of methods for evaluating disease severity (Mini Mental State Exam, Clinical Dementia Rating, disease duration) making comparison difficult. Four studies used a control group consisting in part or entirely of those with subjective cognitive decline (Dukart et al., 2011; Koikkalainen et al., 2016; Möller et al., 2016; Tong et al., 2017). All others consisted of healthy, cognitively normal subjects. Studies also varied widely in their exclusion criteria. Some studies included FTD with concurrent motor symptoms while others excluded these subjects.

### 3.2 bvFTD vs Controls

**Fig. 2:**
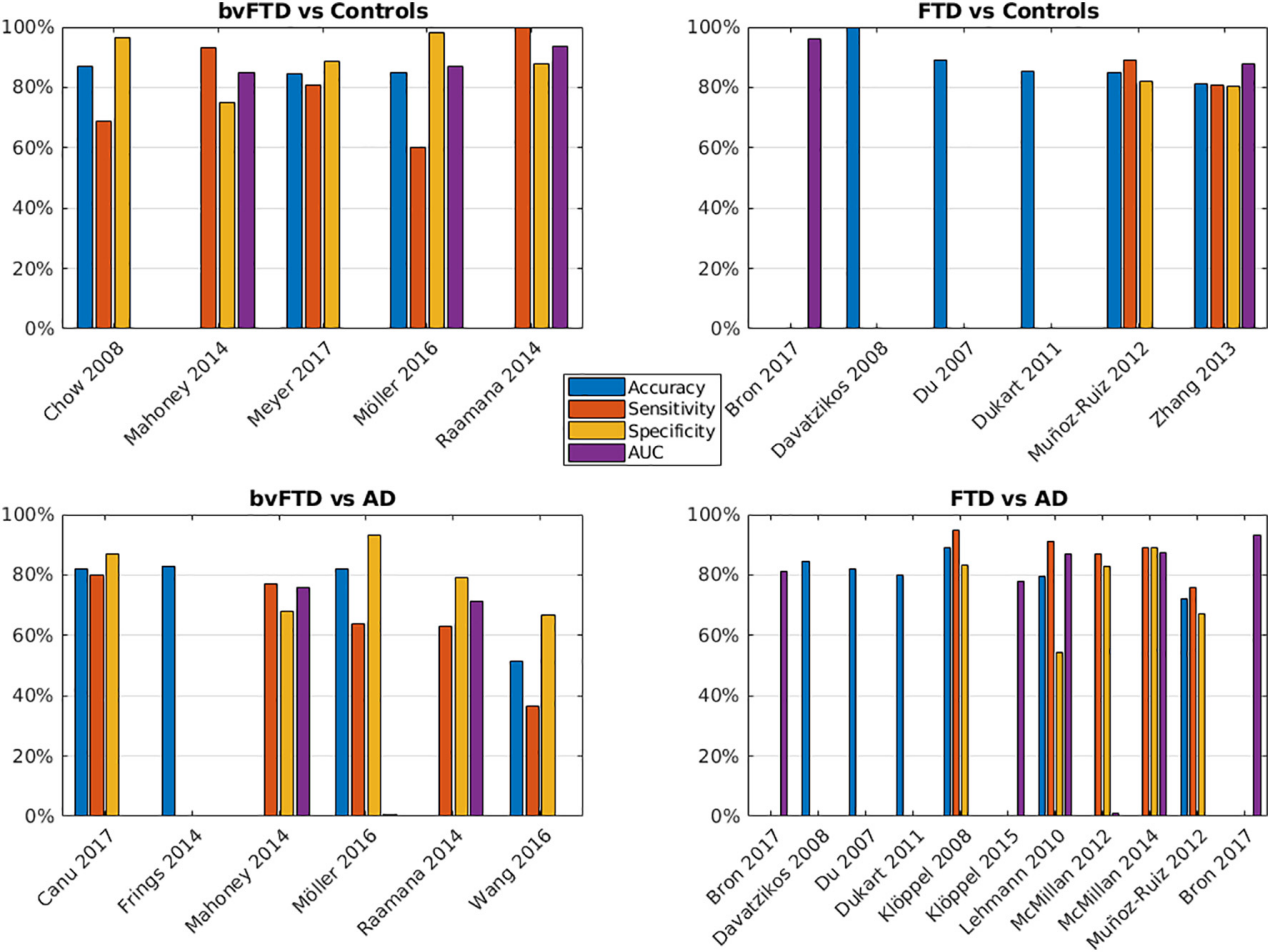
Visual representation of the classification accuracy for the different comparisons (for studies which conducted more than one classification, the best result is shown). a) behavioral variant frontotemporal dementia (bvFTD) vs Controls. b) Frontotemporal dementia (any subtype - FTD) vs Controls. c) bvFTD vs AD. d) FTD (any subtype) vs AD.

Five studies classified bvFTD from a control group (Chow et al., 2008; Mahoney et al., 2014; Meyer et al., 2017; Möller et al., 2016; Raamana et al., 2014) (Table 1 and Fig. 2a). In general studies could distinguish FTD from controls with moderate to high accuracy, although results are heterogeneous. Two studies measured GM concentration with VBM using a SVM classifier. Meyer et al. (2017) achieved highest accuracy, sensitivity and specificity when using a ROI approach (frontal and temporal lobes – 84.6%, 80.7% and 88.5%, respectively), while Möller et al. (2016) reported low sensitivity (60%) but high specificity (98%) with a whole-brain approach. Mahoney et al. (2014) achieved moderate results using radial diffusivity from DTI. The highest result was reported by Raamana et al. (2014) using surface displacements of the left lateral ventricle as inputs to a SVM, using a train/test approach (AUC of 0.938, sensitivity of 100% and specificity of 88%) The result was somewhat lower when using leave-one-out CV (AUC of 0.826, sensitivity of 79, specificity of 87). These results contrast with this study's reported results for other regions (right lateral ventricle and left and right hippocampus) in which sensitivity is low. None of the studies classifying the bvFTD subtype from controls looked at different MRI metrics in combination.

### 3.3 FTD vs controls

Six studies classified a combined group of FTD clinical subtypes from a control group (Table 2 and Fig. 2b), again with overall moderate to high accuracy (Bron et al., 2017; Davatzikos et al., 2008; Du et al., 2007; Dukart et al., 2011; Muñoz-Ruiz et al., 2012; Zhang et al., 2013). Davatzikos et al. (2008) reported 100% accuracy when using GM and WM volumetric features derived from principle component analysis as inputs to an SVM, however this study was small (FTD n = 12) and may not have used a completely independent test set. Very high results were also reported by Bron et al. (2017) when using GM, WM, or supratentorial brain volume with an SVM (AUC 0.95–0.96). This study did not report sensitivity and specificity numbers. In contrast, Zhang et al. (2013) reported poor results using GM or WM volumes and logistic regression in a ROI approach extracted from group differences, but achieved best results using radial diffusivity (accuracy, sensitivity, specificity, and AUC of 81.4%, 80.7%, 80.5%, 0.877, respectively). Two other studies reported moderately high results using various measures of GM structure alone (tensor-based morphometry, volumetry, VBM, cortical thickness) (Du et al., 2007; Muñoz-Ruiz et al., 2012). Only one study (Bron et al., 2017) assessed a multimodal approach (WM volume and fractional anisotropy), which achieved a similar result to that by WM volume alone (AUC 0.95).

### 3.4 bvFTD vs AD

Six studies classified bvFTD from AD (Canu et al., 2017; Frings et al., 2014; Mahoney et al., 2014; Möller et al., 2016; Raamana et al., 2014; Wang et al., 2016) (Table 1 and Fig. 2c). In general, results indicate that this is a much harder task than distinguishing from controls and results are highly variable. Canu et al. (2017) achieved moderately high results using cortical thickness in a random forest approach to distinguish bvFTD from AD (accuracy, sensitivity, and specificity of 82%, 80%, and 87% respectively). These results were not majorly improved when combined with DTI measures. No other study looked at the accuracy of combined MRI metrics. Other studies reported low to moderate accuracy in classifying bvFTD from AD using a range of single metrics including DTI, GM concentration, volumetry, and surface displacements (Frings et al., 2014; Mahoney et al., 2014; Möller et al., 2016; Raamana et al., 2014; Wang et al., 2016).

### 3.5 FTD vs AD

Eleven studies classified FTD (combined clinical subtypes, pathological subtypes, or CSF-defined) from AD (Bron et al., 2017; Davatzikos et al., 2008; Du et al., 2007; Dukart et al., 2011; Klöppel et al., 2015; Klöppel et al., 2008b; Lehmann et al., 2010; McMillan et al., 2014; McMillan et al., 2012; Muñoz-Ruiz et al., 2012; Whitwell et al., 2011) (Table 2 and Fig. 2d). Again, results are highly variable. McMillan et al. (2012) reported highest accuracy when using a combination of GM density and fractional anisotropy (sensitivity, specificity and AUC of 87%, 83%, and 0.938 respectively) when distinguishing CSF-defined FTD and AD using regression, although this study did not use an independent testing set. McMillan et al. (2014) also reported moderately high sensitivity, specificity and AUC (89%, 89%, and 0.874 respectively) to classify CSF-defined FTD and AD when using a combination of cortical thickness and fractional anisotropy in a data-driven approach. In contrast Klöppel et al. (2008b) reported similar numbers using GM volume alone, in a whole-brain approach with an SVM (accuracy, sensitivity, and specificity of 89.2%, 94.7%, and 83.3% respectively), while Whitwell et al. (2011) reported high AUC (0.93) using GM volumes of the temporoparietal cortex and hippocampus. Other studies again reported low to moderate accuracy in classifying FTD from AD with a range of different metrics (Bron et al., 2017; Davatzikos et al., 2008; Du et al., 2007; Dukart et al., 2011; Klöppel et al., 2015; Lehmann et al., 2010; Muñoz-Ruiz et al., 2012).

### 3.6 Multi-class classifications

Several studies attempted a multi-class classification with varying accuracy. Six studies included a three-way classification between FTD, AD, and controls (Bron et al., 2017; Dukart et al., 2011; Kuceyeski et al., 2012; Möller et al., 2015; Raamana et al., 2014; Wang et al., 2016) (Table 3). Kuceyeski et al. (2012) reported the highest accuracy using radial diffusivity, with accuracy and sensitivity of 89.09% and 97.3% but lower specificity (72.22%) using linear discriminant analysis. Results were similar using the LoCo metric, a measurement of the amount of structural network disruption incurred by a GM region for a particular pattern of WM integrity loss (accuracy, sensitivity, and specificity of 87.27%, 91.89%, 77.78% respectively). Four studies conducted a multi-class classification between various dementias and controls (Klöppel et al., 2015; Koikkalainen et al., 2016; Tong et al., 2017; Vemuri et al., 2011) (Table 4). Vemuri et al. (2011) reported moderate sensitivity (84.4%) and high specificity (93.8%) for FTD classification versus all others using whole brain GM density approach and a novel classification approach (referred to as differential-STAND), however they did not have a completely independent test set. Results were considerably lower for other studies (Klöppel et al., 2015; Koikkalainen et al., 2016; Tong et al., 2017).

### 3.7 PPA subtypes

Four studies included classifications of PPA (Bisenius et al., 2017; Chow et al., 2008; Tahmasian et al., 2016; Wilson et al., 2009) (Table 5). Two studies classified each PPA subtype against controls using SVM of GM atrophy, with moderate to high accuracy across studies (accuracy ranged from 84 to 100%) (Bisenius et al., 2017; Wilson et al., 2009). Both studies also classified subtypes against each other, with varying results. Wilson et al. (2009) reported highest accuracy, sensitivity, and specificity (89.1%, 84.4%, 93.8% respectively, AUC of 0.964) to distinguish svPPA from nfvPPA using GM volume and a principal component analysis approach. Results were very high for both studies for lvPPA vs svPPA, while Wilson et al. (2009) achieved highest results for lvPPA vs nfvPPA (accuracy, sensitivity, specificity, AUC of 81.3%, 81.3%, 81.3% and 0.879 respectively). Tahmasian et al. (2016) classified each FTD subtype against a group of all others and AD using GM volume and SVM, resulting in high specificity (97.5% and 94.2%) but very poor sensitivity (50% and 0%) for both svPPA and nfvPPA vs others, while Chow et al. (2008) combined svPPA and nfvPPA subtypes together in a classification from a control group, achieving moderate sensitivity (78.6%) and high specificity (96.7%).

### 3.8 Risk of bias assessment

The results of the QUADAS-2 evaluation are given in Table 6. The patient selection domain was rated as high risk of bias in six studies that had inappropriate exclusion criteria (e.g. exclusion of subjects with abnormalities on structural MRI other than atrophy, such as WM hyperintensities) combined with a case-control design. The index test was rated as high risk of bias in eight studies which did not use separate testing data or used all data to perform ROI selection or dimensionality reduction prior to classification. Two studies were given an unclear risk of bias on this domain. One study was rated as having applicability concerns on the index test domain as it only looked at the overall accuracy of multi-class classification of dementia types.

**Table 6:** QUADAS-2 Evaluation.

Study	Risk of Bias				Applicability concerns		
	Patient selection	Index test	Reference standard	Flow and timing	Patient selection	Index test	Reference standard
Bisenius et al., 2017	Low	Low	Low	Low	Low	Low	Low
Bron et al., 2017	Low	Low	Low	Low	Low	Low	Low
Canu et al., 2017	High	Low	Low	Low	Low	Low	Low
Chow et al., 2008	Low	High	Low	Low	Low	Low	Low
Davatzikos et al., 2008	Low	Unclear	Low	Low	Low	Low	Low
Du et al., 2007	Low	Low	Low	Low	Low	Low	Low
Dukart et al., 2011	High	Low	Low	Low	Low	Low	Low
Frings et al., 2014	Low	High	Low	Low	Low	Low	Low
Klöppel et al., 2008a, Klöppel et al., 2008b	Low	Low	Low	Low	Low	Low	Low
Klöppel et al., 2015	Low	Low	Low	Low	Low	Low	Low
Koikkalainen et al., 2016	Low	Low	Low	Low	Low	Low	Low
Kuceyeski et al., 2012	Low	Low	Low	Low	Low	Low	Low
Lehmann et al., 2010	Low	Low	Low	Low	Low	Low	Low
Mahoney et al., 2014	Low	High	Low	Low	Low	Low	Low
McMillan et al., 2012	Low	High	Low	Low	Low	Low	Low
McMillan et al., 2014	Low	Low	Low	Low	Low	Low	Low
Meyer et al., 2017	Low	Low	Low	Low	Low	Low	Low
Möller et al., 2015	High	High	Low	Low	Low	Low	Low
Möller et al., 2016	Low	Low	Low	Low	Low	Low	Low
Muñoz-Ruiz et al., 2012	High	Unclear	Low	Low	Low	Low	Low
Raamana et al., 2014	Low	Low	Low	Low	Low	Low	Low
Tahmasian et al., 2016	High	Low	Low	Low	Low	Low	Low
Tong et al., 2017	Low	Low	Low	Low	Low	High	Low
Vemuri et al., 2011	Low	High	Low	Low	Low	Low	Low
Wang et al., 2016	Low	Low	Low	Low	Low	Low	Low
Whitwell et al., 2011	Low	High	Low	Low	Low	Low	Low
Wilson et al., 2009	Low	Low	Low	Low	Low	Low	Low
Zhang et al., 2013	High	High	Low	Low	Low	Low	Low

## 4 Discussion

This systematic review provides a summary of studies attempting to classify FTD from non-FTD via morphometric MRI data with the aim to determine its potential for use as a diagnostic aide in clinical practice. Studies included in this review are highly heterogeneous in terms of subject selection, MRI methodology and classification methods, complicating the comparison of accuracy of results. However, overall studies report good levels of accuracy (see Table 7 for a summary of the best performance for each classification), indicating the potential value of MRI morphometry in the diagnosis of FTD.

**Table 7:** Summary of studies with the best performance.

	Name	Sample	Classification	Measures	ROIs	Acc	SS	SP	AUC
bvFTD vs Controls	Raamana et al., 2014	30 bvFTD	SVM	Surface displacements	L lateral ventricle		100	88	0.938
		14 C	Train/test						
bvFTD vs AD	Canu et al., 2017	27 bvFTD	Random forest	Cortical thickness	Best 5 (L inferior parietal, R temporal pole, L isthmus cingulate, R inferior parietal, R precuneus)	82	80	87	
		62 AD							
FTD vs Controls	Davatzikos et al., 2008	12 FTD	SVM	RAVENS-GM and WM volume	PCA	100			
		12 C	LOOCV						
FTD vs AD	McMillan et al., 2014	72 FTD	Linear regression	Combination (Cortical thickness & DTI-FA)	Data-driven		89	89	0.874
		21 AD							
			Train/test						
FTD vs AD & Controls	Kuceyeski et al., 2012	18 FTD	Linear discriminant analysis	DWI-RD	Whole-brain parcellation	89.09	97.30	72.22	
		18 AD							
			LOOCV						
		19 C							
FTD vs other dementias	Vemuri et al., 2011	7 FTD	Differential-STAND	GM density	Whole-brain		84.4	93.8	
			LOOCV						
		48 AD							
		20 DLB							
		21 C4							
nfvPPA vs Controls	Bisenius et al., 2017	6 nfvPPA	SVM	VBM-GM density	Whole-brain	91	88	94	0.94
		20 C	LOOCV						
lvPPA vs Controls	Wilson et al., 2009	16 lvPPA	SVM	GM volume	PCA	100	100	100	1
		115 C	2-level CV						
svPPA vs Controls	Bisenius et al., 2017	17 svPPA	SVM	VBM-GM density	ROI (a priori from meta-analyses)	100	100	100	1
		20 C	LOOCV						
	Wilson et al., 2009	38 svPPA	SVM	GM volume	PCA	100	100	100	1
		115 C	2-level CV						
svPPA vs nfvPPA	Wilson et al., 2009	32 nfvPPA	SVM	GM volume	PCA	89.1	84.4	93.8	0.964
		38 svPPA	2-level CV						
lvPPA vs svPPA	Bisenius et al., 2017	11 lvPPA	SVM	VBM-GM density	Whole-brain	95	100	91	0.93
		17 svPPA	LOOCV						
lvPPA vs nfvPPA	Wilson et al., 2009	32 nfvPPA	SVM	GM volume	PCA	81.3	81.3	81.3	0.879
		16 lvPPA	2-level CV						

FTD could be diagnosed with high accuracy from control groups, with many studies finding accuracies of over 80% or 90% with good sensitivity and specificity. However, most studies include subjects with well characterized patients in which there is significant atrophy, and therefore the added benefit of morphometry is uncertain. Results distinguishing FTD from AD were somewhat poorer. This is unsurprising given that minimal atrophy is expected in control subjects and that there exists overlap in atrophy patterns between FTD and AD (De Souza et al., 2013). Studies which conducted multi-class classifications did not all report specific sensitivity and specificity values for FTD, although Vemuri et al. (2011) reported good sensitivity and specificity (84.4% and 93.8%) in distinguishing FTD from other dementias. Only four studies specifically classified PPAs, generally with moderate to high accuracy. No studies attempted to distinguish bvFTD patients from those with psychiatric disorders, and these two disorders have been shown to be difficult to distinguish clinically (Woolley et al., 2011). However, it is likely that this distinction will be similar to that of control subjects as no atrophy is expected in most psychiatric disorders other than severe and persistent mental illness, such as schizophrenia with chronic psychotropic treatment, that have been linked to subtle volume loss over time (Andreasen et al., 2011).

Most studies have looked at GM atrophy. Fewer studies have used DTI measures, proving mixed results but with some studies suggesting DTI may be more sensitive in the early stages of the disease (Kuceyeski et al., 2012; Zhang et al., 2013). Most studies included in this review only looked at single MRI measures. Hypothetically a multimodal approach combining various MRI modalities such as GM structure and WM integrity should produce more accurate classification than a single modality, as these modalities should provide complimentary information about different aspects of the disease. This is supported by some studies (McMillan et al., 2014; McMillan et al., 2012) while others found no improvement when adding white matter to cortical metrics (Bron et al., 2017; Klöppel et al., 2008a). These differences are likely due to differing patient groups and methodology.

This review focuses on morphometric MRI measures as the majority of studies in this area have focused on morphometry, however a few recent studies have looked at the added benefit of arterial spin labeling MRI or functional MRI (Bron et al., 2017; Tahmasian et al., 2016). This may provide additional discriminative power and is feasible given that these are all MRI sequences that can be performed in the same session.

### 4.1 Comparison to visual MRI reading

Currently, FTD diagnosis is usually assisted via visual reading of MRI scans with or without semi-structured visual rating scales in clinical practice. It is therefore important that an effective MRI morphometry-based classification tool improves on current practices.

Klöppel et al. (2008a) found that radiologists with different levels of experience varied widely in their ability to distinguish pathologically defined FTD from AD on visual reading of MRI (ranges for accuracy, sensitivity, and specificity were 56.8–83.8%, 55.6–83.8%, and 57.9–90.0% respectively) and generally performed poorer than an SVM classifier of GM volume on the same cohort (Klöppel et al., 2008b). Accuracy was positively correlated with the radiologist's level of experience. Koikkalainen et al. (2016) reported much poorer results (overall accuracy of 46.6%, with a sensitivity of 50% for FTD versus others) when using a disease state index classifier on multiple visual rating scales in the multi-class classification of dementia types compared to their morphometric results. In a mixed neuropsychiatric population, visual reading of baseline MRIs by neuroradiologists using visual rating scales reported high specificity (93%) but only moderate sensitivity (70%) in distinguishing bvFTD from non-bvFTD, using clinical diagnosis at two-year follow-up as the gold standard (Vijverberg et al., 2016).

In a cohort of pathologically defined dementia (Harper et al., 2016), unstructured visual assessment by experienced raters resulted in moderate sensitivity (82%) and high specificity (99%) in distinguishing FTD from controls, while moderate sensitivity (74%) and specificity (81%) was achieved when distinguishing FTD from AD. These results are comparable with many of the results obtained from morphometry studies. Semi-structured visual rating scales were found to provide comparatively high sensitivity and specificity in distinguishing FTD from controls (82% and 89% using the medial temporal lobe atrophy (MTA) scale, and 89% and 97% when using an SVM on the results of multiple visual rating scales). Visual rating scales resulted in moderate specificity (81% for an orbito-frontal scale, and 88% when using an SVM on the results of multiple visual rating scales) but low sensitivity (55% and 56%) when distinguishing FTD from AD.

Overall the results from visual radiologists' review appear generally poorer than the best reported results from MRI morphometry studies, indicating the potential usefulness of automated MRI morphometry for improving diagnosis of FTD. However, it is not proven at this point if morphometry outperforms semi-structures visual rating scales (Chow et al., 2011; Harper et al., 2016). It is possible that morphometric approaches could improve diagnostic accuracy in settings where clinicians have less experience in identifying FTD neuroradiological features. (Klöppel et al., 2008a).

### 4.2 Single-subject approach to structural MRI

While there has been major improvement in automated structural MRI processing pipelines over the years, there remain significant methodological challenges to its application at the single-subject level. One of the main limitations to the clinical validity of such methods is the variability with regards to different sites, scanners and repeated image acquisitions. This variability leads to inconsistency in measurements that reduce the accuracy of diagnostic classifications based on subtle differences in atrophy or other morphometric measures (Potvin et al., 2017). While a comparison of the performance of the different currently available processing pipelines is beyond the scope of this paper, the ideal MRI processing pipeline must perform robust registration and tissue contrast normalization to achieve precise cortical and subcortical segmentation across different scanners. It should further be able to perform intra-subject registration to measure subtle brain changes over time. Being able to compare subjects to a large database of healthy controls across ages, sex and education level is also of significant benefit (Potvin et al., 2017).

### 4.3 Limitations

Studies included in this review are highly heterogeneous in terms of population demographics and methodology. These issues are similar to those regarding the diagnostic classification of AD (Falahati et al., 2014; Rathore et al., 2017).

Studies varied considerably on the subjects they included. Studies using small homogenous samples may result in the overfitting of data. A major issue with studies is the inclusion of well-characterized subjects that tend to be at a later disease stage and therefore may find higher accuracy because brain changes are more substantial and easier to differentiate. Ideally studies need to include patients in the earliest stages of the disease when diagnoses are ambiguous, such as the naturalistic symptom-based inclusion approach taken by the Late-Onset Frontal lobe study (Krudop et al., 2014). Many studies grouped FTD clinical variants together in analysis. Others have indicated that this may lead to the language variants driving the classification resulting in higher performance (Möller et al., 2016). Several studies conducted a group-level analysis and then used the significant regions from this analysis in their classification. This will reduce the generalizability of the results as the regions used may likely be biased to the specific group of patients included in the study. For these reasons, results may be artificially high. Most studies utilized a cross-validation approach, where *k* subjects are sequentially left out of the training group, while others split the subjects into separate training and testing sets. Ideally studies should also validate classifiers on a separate independent cohort. It is likely that this would result in lower accuracy than the numbers reported in several of the studies reviewed here, given the methodology used.

Studies also differed in the metrics used to report results. Here we have reported the most common metrics across studies (accuracy, sensitivity, specificity, and AUC). Some studies did not report sensitivity/specificity but only accuracy or AUC. While useful, these metrics are not sufficient on their own. As only a small number of studies reported balanced accuracy those numbers are not reported here.

Studies included in this review focused predominantly on sporadic FTD. A significant proportion of FTD cases are monogenic in nature (i.e., they are caused by an autosomal-dominant genetic mutation). To our knowledge there have been no published studies of single-subject morphometric MRI classification in the presymptomatic or early symptomatic stages of monogenic FTD. Studies in this population would be of interest to identify biomarkers of the preclinical or early clinical stage that would be a great benefit for future disease-modifying clinical trials of FTD. In addition, it remains to be determined how accurate FTD MRI biomarkers developed with sporadic FTD cohorts would fare in a population of genetic FTD given their well-documented less typical atrophy patterns extending beyond frontal and anterior temporal areas (Rohrer et al., 2015; Whitwell et al., 2015; Whitwell et al., 2012).

Most importantly, few published studies have attempted to apply machine learning derived diagnostic classifiers to real-life clinical settings at the individual level. This is a crucial step given that clinical populations are more heterogenous than well-characterize cohorts from large-scale imaging studies. For instance, pre-existing brain changes (e.g., past cerebro-vascular accident) and co-morbidities (e.g., alcohol use disorder) are commonly seen in memory clinics but are often not represented by the training sets of these studies. Only one study identified in this review attempted to replicate the typical population of a memory clinic (Klöppel et al., 2015). Although this comes with significant challenges and lower accuracy than in the training set (Klöppel et al., 2015), it is an essential step before recommending the clinical use of these algorithms.

Limitations of this systematic review include the possibility of incomplete retrieval of relevant papers, however more than one search engine was used and reference lists of included papers were reviewed for additional relevant papers, so this should be minimal. As only published studies were included in this review there is the potential for publication bias. The main biases identified in the included studies were the exclusion of subjects with abnormalities other than atrophy on structural MRI and the lack of an independent testing set.

### 4.4 Future directions

In order to translate morphometric tools for FTD in clinical practice, it will be crucial to validate the use of automated morphometric MRI methods in a naturalistic mixed neuropsychiatric population, such as the distinction of those presenting with FTD-like symptoms at baseline into those ultimately diagnosed with FTD versus those not. Future studies should validate MRI automated morphometry methods in a mixed cohort of early disease stage patients, using final diagnosis (and ideally when available proven pathology at autopsy) as a gold standard. Larger multi-site datasets will also be important to develop deep learning approaches for categorical diagnostic classification, disease course prediction and to build models that could predict pathological subtypes in vivo (Perry et al., 2017). Morphometry could also improve practice by identifying data-driven subtypes with clinically relevant differences in symptom profile or prognosis (Ranasinghe et al., 2016). The methodology needs to be feasible for use in clinical practice; a straight-forward process that is not time consuming and is easy to interpret is needed, and it needs to be applicable across scanner types and centers. This type of method may be especially helpful for those clinicians with less experience diagnosing FTD, such as community hospitals and primary care physicians that do not have easy access to specialty FTD clinics. In addition to leading to earlier diagnosis and improved prognosis clinically, morphometric biomarkers could potentially improve patient selection and reduce required sample sizes in clinical trials (Pankov et al., 2016), which would accelerate drug discovery.

## 5 Conclusions

Automated morphometric MRI has potential to improve the diagnosis and prognosis of early stage FTD in clinical practice. Current evidence provides good support for its ongoing development. The inclusion of 3D-T1 MRI sequences in clinical imaging protocols would facilitate the development of these tools, and eventually the integration of these methods in practice. However, more studies that use rigorous methodology and prospectively validate findings in independent real-life cohorts are needed before this method could be recommended in clinical practice.

## Footnotes

1 For FTD vs AD classifications, sensitivity is defined as the proportion of correctly classified FTD subjects and specificity as the proportion of correctly classified AD subjects.
